# Étude comparative de l’innocuité de deux vaccins commerciaux contre la babésiose canine provoquée par *Babesia canis*

**DOI:** 10.1051/parasite/2011184311

**Published:** 2011-11-15

**Authors:** L. Freyburger, L. Lemaitre, C. Médaille, F. Oberli, L. Fanchon, P. Bergamo

**Affiliations:** 1 École Nationale Vétérinaire d’Alfort Maisons-Alfort France; 2 Merial S.A.S. Lyon France; 3 Laboratoire Vébiotel Arcueil France

**Keywords:** Pirodog^®^, Nobivac Piro^®^, inocuité, vaccin, babésiose canine, piroplasmose, Pirodog^®^, Nobivac Piro^®^, safety, vaccine, canine babesiosis, piroplasmosis

## Abstract

L’innocuité de deux vaccins commercialisés en France contre la babésiose canine – Nobivac Piro^®^ (NP) et Pirodog^®^ (P) – a été étudiée. Leur impact local, général et biochimique a été comparé, en conditions expérimentales maîtrisées, sur un groupe témoin (T) et deux groupes vaccinés deux fois à 21 jours d’intervalle. Tous les chiens ont présenté une réaction locale modérée. Cependant, le groupe NP a présenté une réaction locale significativement plus intense que le groupe P. Ceci est objectivé par les paramètres cliniques et biologiques. Aucune différence statistiquement significative n’est mise en évidence entre les évolutions des groupes P et T.

## Abstract (en)

The safety of two vaccines available on the French market against canine babesiosis – Nobivac Piro^®^ (NP) and Pirodog^®^ (P) – have been evaluated. Their local, general and biochemical impacts have been compared in a controlled experimental study. Three groups were used: a control group (T) and two groups vaccinated twice at 21 days interval. All dogs presented moderate local reaction. However, either clinical and biological parameters showed that the NP group presented a significantly more intense reaction at the injection site compared to the P group. No statistical difference has been revealed between the groups P and T evolutions.

## Introduction

La babésiose canine est une maladie infectieuse systémique, résultant de l’inoculation de parasites du genre *Babesia* par des tiques. Elle a été décrite pour la première fois en Italie à la fin du 19^ème^ siècle. Trois espèces de babésies dites de “grande forme” infectent le chien : *Babesia canis*, *Babesia rossi* et *Babesia vogeli*. *B. canis* est considérée comme la principale espèce présente en France (Beugnet & Marie, 2009; Bourdoiseau, 2006), alors que *B. vogeli* est présente dans d’autres pays méditerranéens et *B. rossi* exclusivement dans le Sud de l’Afrique. Le tableau clinique de la babésiose regroupe un syndrome fébrile et hémolytique, pouvant se compliquer et être responsable de la mort de l’animal infecté. *B. vogeli* est responsable d’une forme modérée de babésiose en comparaison de la forme classique à *B. canis*, tandis que *B. rossi* est à l’origine de formes graves.

Dès le début du 20^ème^ siècle, Nocard émet l’hypothèse qu’une réponse immunitaire protectrice pouvait être induite chez le chien et aboutir à un état d’immunité favorable à la guérison, ainsi qu’à l’absence de nouvelle contamination de l’animal dans les mois qui suivent la guérison (Schetters, 2005). Les antigènes parasitaires solubles, découverts dans le plasma sanguin des chiens naturellement infectés (Sibinovic *et al.*, 1967), ont été parmi les premiers antigènes vaccinaux utilisés. Ils assurent une immunité croisée vis-à-vis de différentes espèces de *Babesia* et confèrent une couverture vaccinale meilleure que celles induites par des souches de babésies vivantes atténuées (Schetters *et al.*, 2001). Ils ne présentent par ailleurs aucun pouvoir pathogène résiduel.

Ces antigènes parasitaires solubles entrent actuellement dans la composition de deux vaccins commercialisés en France induisant un état d’immunité dirigée contre *B. canis* : Novibac Piro^®^ (Intervet) et Pirodog^®^ (Merial). Les adjuvants de ces vaccins sont constitués de saponines, glycosides naturels dérivés de stéroïdes ou triterpènes (Skene & Sutton, 2006; Sun *et al.*, 2009). Les plus connues ont été extraites du *Quillaja saponaria* par Molina au début des années 1990 (Kensil *et al.*, 1991). Les principaux effets secondaires des saponines sont des réactions inflammatoires locales au site d’injection (Waite *et al.*, 2001), caractérisées par une douleur d’intensité variable à l’injection et une induration potentiellement persistante. Chez le chien, l’inflammation peut être suivie par certains marqueurs biologiques : la protéine C réactive (indicateur majeur d’inflammation aiguë), le fibrinogène et l’haptoglobine (protéines mineures de l’inflammation), la vitesse de sédimentation, le TNFα et la caractérisation des protéines sériques par électrophorèse.

En France, l’utilisation de ces deux vaccins en pratique courante semble mettre en évidence une différence d’innocuité. Afin d’infirmer ou de confirmer cette différence, nous avons comparé en conditions contrôlées l’innocuité des deux préparations vaccinales présentant par ailleurs une efficacité validée par les études précédant l’autorisation de mise sur le marché. L’étude, réalisée en double aveugle, a permis de suivre l’évolution de paramètres cliniques et biochimiques afin d’objectiver l’apparition d’une réaction inflammatoire post-vaccinale.

## Matériel, méthodes et animaux

### Préparations pour immunisation

Pirodog^®^ (lot commercial L257544) est un vaccin lyophilisé; il contient des antigènes solubles inactivés et concentrés de *B. canis* en quantité suffisante pour obtenir un titre en anticorps après vaccination supérieur à 1/160 chez 75% des chiens. Le solvant contient de la saponine à 0,5 mg/mL dans 1 mL d’eau pour préparation injectable.

Nobivac Piro^^®^^ (lot commercial A006A09) est également un vaccin lyophilisé. Il contient entre 301 et 911 unités de masse totale d’antigènes solubles parasitaires inactivés issus de culture de *B. canis* et de *B. rossi*. Le solvant contient de la saponine à 0,25 mg (entre 0,225 mg et 0,275 mg) dans 1 mL d’eau pour préparation injectable.

Le groupe témoin a reçu des injections de 1 mL d’eau pour préparation injectable.

### Animaux

L’étude est réalisée chez 20 Beagles mâles. Ils sont âgés de 27 à 34 semaines le jour de la primo-injection. Les animaux inclus n’ont pas reçu préalablement de vaccin contre la babésiose. Tous les chiens subissent un traitement antiparasitaire externe une semaine avant chaque immunisation. L’hébergement a lieu en box individuel, sans contact possible entre les chiens.

### Protocoles de vaccination

Les animaux sont répartis aléatoirement en trois groupes recevant respectivement deux injections à J0 et J21 :

- le groupe témoin : eau pour préparation injectable;
- le groupe P : vaccin Pirodog^®^ à J0 et J21;
- le groupe NP : vaccin Nobivac Piro^®^ à J0 et J21.

Les injections sont réalisées conformément aux indications de l’AMM (1 mL, voie sous-cutanée), dans la zone interscapulaire tondue pendant la semaine précédant la vaccination

### Examens et scores cliniques

Les animaux ont fait l’objet d’un examen clinique, par le même opérateur, avant chaque injection, puis à 8 heures (8H), 24 heures, 48 heures et 72 heures. L’examen comprend la prise de température rectale, l’évaluation de l’état général, de la douleur à la palpation, de la taille du pli de peau, de la présence d’une tuméfaction, de la chaleur cutanée et du prurit. La prise d’aliment est contrôlée par pesée des aliments distribués et des aliments non consommés.

Les données cliniques permettent d’établir un score clinique global général (SCGG) et un score clinique global local (SCGL) (Tableau I). Le SCGG correspond à la somme des pondérations de la température rectale et de l’état clinique général. Les températures rectales ont été groupées et un score a été attribué à chaque groupe (hypothermie, normothermie, hyperthermie modérée et hyperthermie sévère), de même pour l’état général (bon état général, apathie, abattement sévère). Le SCGL comprend les paramètres pondérés suivants : douleur à la palpation (absence ou présence), tuméfaction (absence, palpable, mesurable ≤ 2 cm, mesurable > 2 cm), chaleur cutanée (absence ou présence), prurit au site d’injection (absence ou présence), autres (tous signes potentiellement liés à la vaccination objectivés). Plus les SCGL sont élevés et différents de 0, plus ils signent une réaction locale importante.

**Tableau I.** Grille de cotation des Score clinique global général (SCGG) et Score clinique global local (SCGL).

	Critères	0	1	2	3
SCGG	Température rectale	37,0 °C ≤ T ≤ 39,5 °C	39,5 °C ≤ T ≤ 40,5 °C	T ≥ 40,5 °C	T ≤ 37,0 °C
	État général	Bon	Apathie		Abattement
SCGL	Douleur à la palpation	Absence	Présence		
	Tuméfaction	Absence	Palpable	Mesurable ≤ 2 cm	Mesurable > 2 cm
	Chaleur cutanée	Absence	Présence		
	Prurit	Absence	Présence		
	Autres signes liés à la vaccination	Absence	Présence		

### Prélèvements sanguins et analyses biologiques

Les prélèvements sanguins sont effectués sur tube sec et tube EDTA K3, en vue d’étudier la réponse inflammatoire grâce aux analyses suivantes effectuées au laboratoire Vébiotel (Arcueil, 94) excepté le dosage du TNFα réalisé chez RD-Biotech (Besançon, 25) :

- dosage des protéines totales et électrophorèses des protéines sériques;
- dosage de l’haptoglobine – Konelab 30 I (ThermoElektron), réactif Haptoglobine (Thermo) –, technique validée chez le chien (Weidmeyer & Solter, 1996), de la protéine C réactive – dosage immunoturbidimétrique sur Konelab 30 I (ThermoElektron), réactif CRP plus Thermo –, technique validée chez le chien (Kjelgaard- Hansen *et al.*, 2003), du TNFα – Quantikine^®^ canine TNFα immunoassay, CATA00, R&D Angleterre –, du fibrinogène – dosage turbidimétrique UV sur Konelab 30 I (ThermoElektron), réactif Sobioda;
- mesure de la vitesse de sédimentation (méthode de Westergren) à une heure (VS1) et à deux heures (VS2).

Les résultats des analyses des protéines totales, du fibrinogène, de l’haptoglobine et du TNFα, d’une part, et les électrophorèses des protéines sériques, d’autre part, n’ont donné aucune variation significative dans notre étude et ne seront donc pas détaillés dans la suite de l’article.

## Résultats

### Paramètres cliniques

#### • Score clinique global général

Les scores ne diffèrent pas et ne démontrent aucune variation par rapport à l’état général global initial avant immunisation. Aucune différence significative n’a été relevée entre les groupes NP, P et témoin (résultats non présentés), ni en comparant les groupes vaccinés au groupe témoin consécutivement aux deux injections.

#### • Température rectale

**Figure 1.**
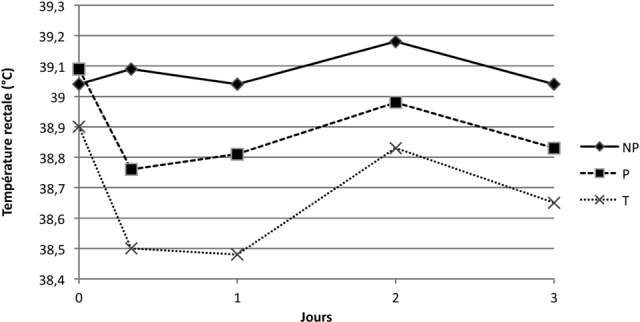
Moyenne des températures rectales (°C) suite à la primoimmunisation des groupes NP, P, et T.

**Figure 2.**
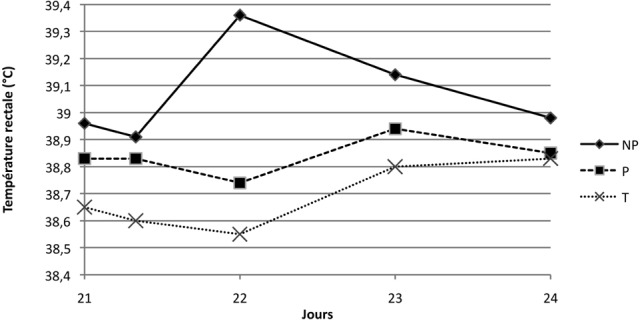
Moyenne des températures rectales (°C) suite à la seconde immunisation des groupes NP, P et T.

Les températures rectales restent dans les normes physiologiques. Suite à la première immunisation, il n’a pas été observé de différence significative entre les groupes en ce qui concerne l’évolution dans le temps de la température (p = 0,33) (Figure 1); ce n’était en revanche pas le cas après la seconde immunisation, avec une différence significative entre le groupe NP et les autres groupes. En effet, la température moyenne des chiens du groupe NP augmente de 0,5 °C entre J21 + 8H et J22 pour atteindre une valeur moyenne de 39,4 °C, alors que celles des chiens des groupes P ne varient que de l’ordre de 0,1 °C pour atteindre 38,7 °C (p = 0,001) (Figure 2).

#### • Prise d’aliments

Aucune différence de prise d’aliments n’est mise en évidence entre les groupes NP et P, au cours des trois jours de suivis post-immunisation que ce soit à la primo-immunisation (p = 0,46) ou au rappel (p = 0,64).

#### • Score clinique global local

##### Première immunisation à J0

Le groupe témoin présente un SCGL nul. Le groupe NP présente un pic compris entre 8 et 24 heures postimmunisation à 1,5, une phase de stabilisation jusqu’à J2, puis une diminution légèrement inférieure à 1 (0,88) à J3 post-immunisation. Il est significativement différents du groupe témoin (p = 0,005 pour NP, par le test ANOVA).

**Figure 3.**
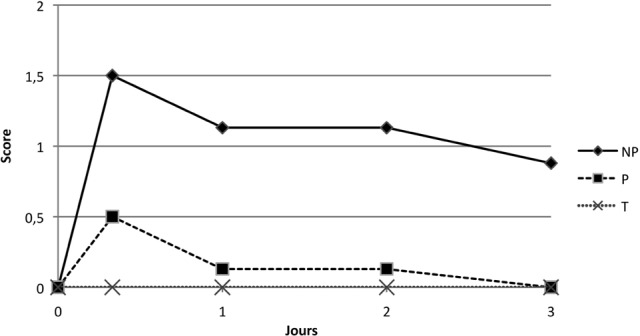
Moyenne des SCGL suite à la première vaccination des groupes NP, P et T.

Le SCGL du groupe P augmente à J0 + 8H à une valeur faible de 0,5 puis diminue rapidement pour être nul au troisième jour post-immunisation. Les groupes témoin et P ne diffèrent pas pour ce paramètre (p = 0,62 par le test ANOVA). Le SCGL est significativement moins élevé dans le groupe P que dans le groupe NP (p = 0,007) lors de la primo-vaccination (Figure 3).

L’inflammation locale à l’issue de l’injection de P est inférieure au groupe ayant reçu NP en primo-immunisation, avec un écart de moyenne de une unité en score de SCGL entre le groupe P et le groupe NP, au pic d’inflammation à J0 + 8H (Figure 3). Cette réaction locale est moins durable pour le groupe P (24 heures postimmunisation) par rapport au groupe NP (trois jours pour diminuer en dessous de 1, sans revenir à l’origine).

##### Seconde immunisation à J21

Le groupe témoin présente un SCGL nul. Pour les autres groupes, une augmentation de la moyenne du SCGL dans les trois jours qui suivent l’injection de rappel est clairement mise en évidence. Il existe une différence statistiquement significative sur les trois jours de suivi entre le groupe témoin et le groupe NP (p = 0,02).

**Figure 4.**
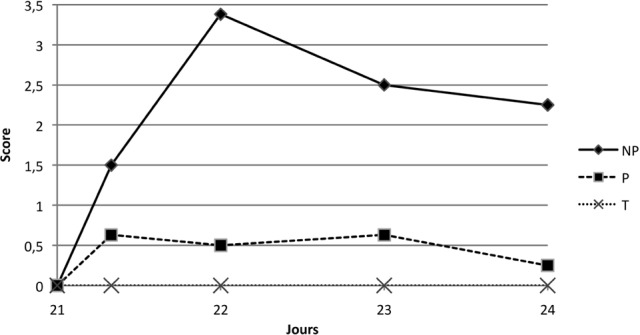
Moyenne des SCGL suite à la seconde immunisation pour les groupes NP, P et T.

Pour le groupe NP, la valeur du SCGL augmente de façon notable après l’injection de rappel. Le pic est atteint à J22, avec un score de 3,38 (Figure 4). Cette valeur est 2,25 fois supérieure au pic atteint à J0 + 8H, suite à la première immunisation. Le groupe P évolue de façon quasiment identique à la première immunisation, avec un pic huit heures après injection, légèrement supérieur pour le rappel (0,67 à J21 + 8H *versus* 0,5 à J0 + 8H). L’évolution de ce paramètre au cours du temps est différente d’un groupe à l’autre (p = 0,02). Lorsque le groupe P atteint un plateau à J21 + 8H, le groupe NP continue à augmenter jusqu’à J22 (Figure 4). Ainsi, en moyenne, à J22, J23 et J24, le groupe NP évolue de façon significativement différente du groupe P (p = 0,0002).

La réaction locale dans le groupe NP est exacerbée lors de la seconde immunisation, alors qu’elle ne l’est pas pour les groupes P. L’impact local de la seconde immunisation dans le groupe P n’est statistiquement pas différente de celle du groupe témoin (p = 0,06).

#### • Mesure du pli de peau

Au cours des trois jours suivant la première immunisation, bien que l’évolution dans le temps du pli de peau ne soit pas différente entre les trois groupes (p = 0,11), l’intensité de cette variation est moindre pour le groupe P par rapport aux groupe NP (p = 0,0001).

**Figure 5.**
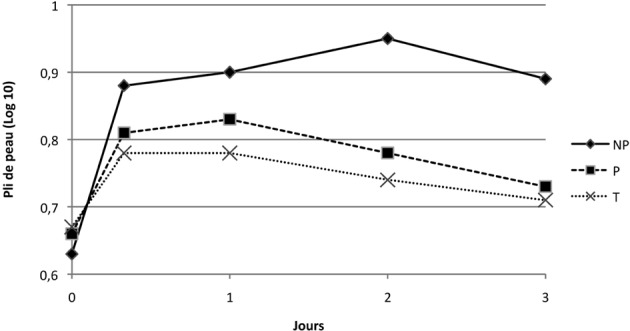
Moyenne des mesures de plis de peau (transformées en log 10) suite à la primo-vaccination des groupes NP, P et T.

La moyenne de l’épaisseur du pli de peau double, en effet, pour le groupe NP (de 4,25 mm à 9 mm à J2) contrairement au groupe P, pour lequel l’augmentation est plus modérée et moins persistante (6 mm à J2, le pic étant obtenu à J1 avec 6,75 mm) (Figure 5).

**Figure 6.**
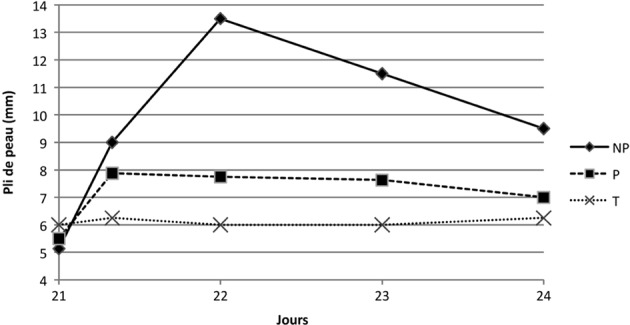
Moyenne des mesures de plis de peau suite au rappel des groupes NP, P et T.

Consécutivement au rappel, l’évolution du pli de peau dans le temps dans les différents groupes varie de façon significativement différente dans les trois jours post-immunisation (p = 0,0027). En effet, l’évolution du groupe P atteint un maximum pour la moyenne des mesures de pli de peau de 7,88 mm puis une régression progressive sur trois jours. Le groupe NP, dont le maximum est de 13,5 mm à J22 évolue de façon différente que le groupe P (p < 0,0001) (Figure 6).

### Paramètres biologiques

#### • CRP

Entre J0 et J8, la CRP du groupe témoin restent stables à un niveau basal de l’ordre de 2,5 mg/L et dans les limites de l’intervalle de référence (< 10 mg/L). La CRP du groupe NP atteint un pic à J1 de 11,3 mg/L. Le groupe P présente aussi une augmentation de la CRP avec un pic à J1 à 9,8 mg/L. La CRP du groupe NP est significativement différente de la CRP du groupe P à J2 et J3. Cela se traduit par une diminution plus rapide à J2 et J3 du taux de CRP dans le groupe P (6 mg/L contre 11,1 mg/L pour NP à J2 et 5,3 mg/L contre 8,3 mg/L à J3). Les concentrations sériques des différents groupes se normalisent en une semaine.

**Figure 7.**
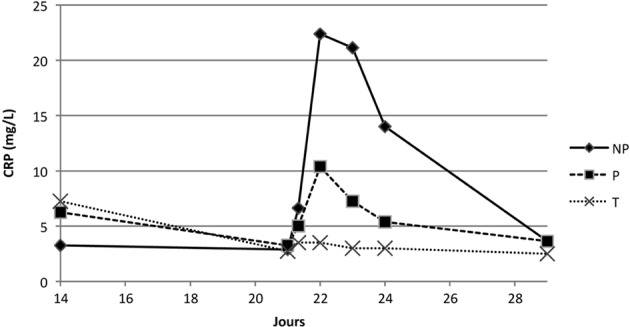
Moyenne des dosages de la CRP suite à la seconde immunisation des groupes NP, P et T.

Suite à l’injection de rappel à J21, les valeurs de CRP obtenues pour le groupe témoin et le groupe P restent similaires à celles de la primo-immunisation. Le groupe NP évolue de façon beaucoup plus forte que le groupe P (Figure 7). Pour NP, la CRP est multipliée par 7 entre J21 (valeur basale de 2,9 mg/L) et J22 (22,4 mg/L). À cette date, la CRP dépasse la valeur physiologique (10 mg/L). Cette cinétique est concordante, selon la sensibilité de la technique du laboratoire d’analyse, avec un processus inflammatoire. Les valeurs diminuent progressivement en une semaine, et retrouvent le seuil basal à J29. Sur l’évolution du paramètre au cours du temps, une différence significative est relevée entre les groupes NP et P (p = 0,0007).

#### • Vitesse de sédimentation (VS1 et VS2)

Le groupe témoin présente des VS1 dans l’intervalle physiologique tout au long du suivi de la primo-immunisation. La valeur maximale du groupe témoin est obtenue à J2 avec 3 mm/h (seuil à 2 mm/h). Suite à la première immunisation, les moyennes des VS1 pour les groupes NP et P dépassent la valeur seuil (2 mm/h) entre J2 et J3, pour une normalisation à J8. Néanmoins, l’amplitude de la variation est très différente entre le groupe NP et le groupe P. Ce dernier varie de façon modérée avec VS1 qui évolue à 4,8 mm/h à J3, alors que le groupe NP culmine à 9,4 mm/h à J3. Ces évolutions sont retrouvées au niveau de la VS2. La VS2 maximale (norme de 4 mm/h) est obtenue à J2, avec 20 mm/h pour le groupe NP et 11,3 mm/h pour le groupe P. Le groupe témoin reste à des valeurs faibles, avec un maximum de 7 mm/h à J2. Toutefois, aucune différence significative concernant l’évolution de ces paramètres au cours du temps selon les groupes n’a été observée. Les évolutions de VS1 et VS2 mettent en évidence un état inflammatoire modéré suite à la première injection vaccinale.

**Figure 8.**
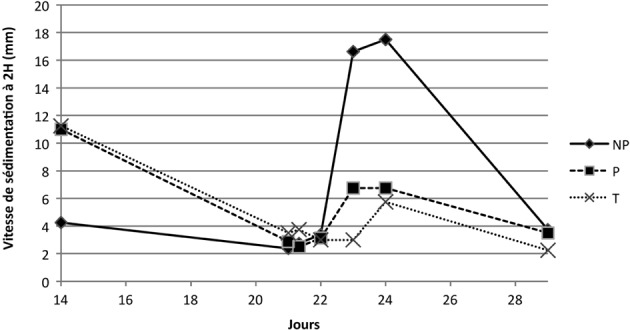
Vitesse de sédimentation des groupes NP, P et T suite à la seconde immunisation.

Suite à la seconde injection, l’évolution des vitesses de sédimentation dans l’ensemble est similaire, avec toutefois des valeurs absolues plus faibles pour l’ensemble des groupes. Seul le groupe NP montre une modification biologiquement significative avec 17 mm/h pour la VS2 entre J23 et J24 (Figure 8). À ces dates, les valeurs de VS2 sont significativement (p = 0,015) moins élevées pour le groupe P (6,75 mm/h).

Suite à la primo-immunisation, les scores cliniques locaux mettent en évidence un état inflammatoire qui ne se retrouve pas au niveau systémique dans cette étude. Parallèlement, seules les valeurs de la CRP et des VS tendent à biologiquement souligner les constatations cliniques. Ces observations se confirment lors de la seconde immunisation.

## Discussion

Le profil d’innocuité des deux vaccins anti-babésiens actuellement disponibles sur le marché semblant présenter des différences, nous avons souhaité les comparer en conditions contrôlées. Nous ne nous sommes pas intéressés ici aux paramètres d’efficacité qui ont fait l’objet d’études précédant l’autorisation de mise sur le marché.

L’étude comparative d’innocuité, menée en double aveugle, a porté essentiellement sur le suivi des paramètres biologiques (protéines sériques totales, haptoglobine, CRP, TNFα, fibrinogène, électrophorèse des protéines sériques, vitesse de sédimentation) et l’observation des éventuelles réactions locales et générales post-vaccinales.

Le groupe NP induit des modifications locales et biologiques plus importantes, et généralement significativement différentes, par rapport au groupe P (SCGL, température rectale, pli de peau, cinétique d’évolution de la CRP). Pour ces paramètres locaux, les groupes P et témoin ne présentent pas de différence statistiquement significative, à la différence du groupe NP *versus* témoin.

Ces observations sont cohérentes avec les mentions de la section 6 (Effets indésirables) du résumé des caractéristiques produit (Agence Européenne du Médicament, 2009). Il est en effet mentionné que “*les réactions post-vaccination couramment rapportées sont un oedème diffus et/ou un nodule induré, douloureux, au point d’injection*”. Nous n’avons cependant pas mis en évidence dans notre étude de réaction douloureuse.

L’étude n’a révélé qu’un impact modéré de la vaccination sur l’état général des chiens, sans différence significative entre les groupes. Néanmoins, en 2007, le rapport de pharmacovigilance de l’Institut Vétérinaire Fédéral Suisse (IVI) rapporte des intolérances postvaccinales (dégradation de l’état général, apathie, fièvre, douleur généralisée) avec un vaccin anti-babésien canin, correspondant au vaccin NP testé dans notre étude (Muntener *et al.*, 2007). L’incidence de ces intolérances a été évaluée sur l’année 2006 à 1%. Une mise en garde a d’ailleurs été ajoutée dans les mentions légales de la spécialité. Les chiens suivis dans notre étude n’ont pas présenté d’altération majeure de l’état général. Un tel impact pourrait ne pas avoir été mis en évidence étant donné le faible nombre de chiens inclus. Une moindre sensibilité des beagles peut aussi être envisagée.

D’une manière générale, les réactions vaccinales, locales ou générales, peuvent être liées à l’effet proinflammatoire des adjuvants. Les deux vaccins antibabésiens contiennent des adjuvants appartenant à la famille des saponines. L’étude de Parra (Parra *et al.*, 2007) indiquait chez presqu’un quart des chiens recevant une spécialité adjuvée à base de saponines la présence d’effets systémiques (anorexie, apathie). Schetters (Schetters *et al.*, 2001; Schetters *et al.*, 2006) a montré que la dose de saponine avait un impact systémique d’autant moins intense que la dose était réduite. Ces études mentionnent également des effets secondaires locaux (douleur à l’injection chez environ 40% des chiens, oedème localisé se résorbant en cinq jours chez plus de 15% des chiens dans l’étude de Parra (Parra *et al.*, 2007)). Giunchetti rapporte dans une étude (Giunchetti *et al.*, 2007) l’apparition chez plusieurs chiens ayant reçu des saponines, d’un nodule inflammatoire de taille supérieure à 4 cm^2^ au point d’inoculation, sans atteinte de l’état général. La différence observée dans la présente étude, entre les effets locaux induits par les deux vaccins testés, pourrait être liée à une différence de composition et/ou de concentration d’adjuvant, même s’ils appartiennent à la même famille des saponines.

Concernant les paramètres biologiques, les études des valeurs usuelles (Kuribayashi *et al.*, 2003) et le dosage des marqueurs de l’inflammation chez le chien (McGrotty *et al.*, 2003; Nakamura *et al.*, 2008) ne fournissent pas d’étalon ou de critère cardinal affection par affection (Nakamura *et al.*, 2008). Il semble que les inflammations locales (par exemple une rhinite) ne déclenchent pas d’augmentation notable de la concentration sanguine en CRP contrairement à des inflammations profondes de type bronchite. Les protéines mineures de l’inflammation sont augmentées lors d’affections inflammatoires généralisées chroniques (comme la leishmaniose); l’haptoglobine peut être diminuée dans les cas de babésiose clinique par liaison à l’hémoglobine libre (Ulutas *et al.*, 2005) et lors de prise de corticoïdes. Dans notre étude, une variation systémique a été mise en évidence pour la CRP et la vitesse de sédimentation. Cette évolution est cohérente avec un état inflammatoire modéré, en particulier avec le vaccin NP. Toutefois, aucun des paramètres étudiés dans cette étude ne se révèle être un bon candidat comme facteur de suivi de l’inflammation consécutive à la vaccination anti-babésiose. Dans le cadre de cette étude, le suivi clinique des réactions locales inflammatoires consécutives à la vaccination étudiée apparaît plus sensible que l’étude des paramètres biologiques caractérisant l’inflammation.

Ces données mettent donc en évidence une différence d’innocuité entre les vaccins Nobivac Piro^®^ et Pirodog^®^, et objectivent ainsi les observations de terrains.
